# Comparison of Porcine Small Intestinal Submucosa versus Polypropylene in Open Inguinal Hernia Repair: A Systematic Review and Meta-Analysis

**DOI:** 10.1371/journal.pone.0135073

**Published:** 2015-08-07

**Authors:** Xin Nie, Dongdong Xiao, Wenyue Wang, Zhicheng Song, Zhi Yang, Yuanwen Chen, Yan Gu

**Affiliations:** 1 Department of General Surgery, Shanghai Ninth Hospital affiliated to Shanghai JiaoTong University School of Medicine, and Hernia and Abdominal Wall Surgery Center of Shanghai JiaoTong University, Shanghai, China; 2 Department of Urology Surgery, Shanghai Ninth Hospital affiliated to Shanghai JiaoTong University School of Medicine, Shanghai, China; University of Arizona, UNITED STATES

## Abstract

**Background:**

A systematic review and meta-analysis was performed in randomized controlled trials (RCTs) to compare porcine small intestinal submucosa (SIS) with polypropylene in open inguinal hernia repair.

**Method:**

Electronic databases MEDLINE, Embase, and the Cochrane Library were used to compare patient outcomes for the two groups via meta-analysis.

**Result:**

A total of 3 randomized controlled trials encompassing 200 patients were included in the meta-analysis. There was no significant difference in recurrence (P = 0.16), hematomas (P = 0.06), postoperative pain within 30 days (P = 0.45), or postoperative pain after 1 year (P = 0.12) between the 2 groups. The incidence of discomfort was significantly lower (P = 0.0006) in the SIS group. However, the SIS group experienced a significantly higher incidence of seroma (P = 0.03).

**Conclusions:**

Compared to polypropylene, using SIS in open inguinal hernia repair is associated with a lower incidence of discomfort and a higher incidence of seroma. However, well-designed larger RCT studies with a longer follow-up period are needed to confirm these findings.

## Introduction

Inguinal hernia is a common type of abdominal wall defect, with more than 990,000 abdominal wall hernia repair surgical procedures performed in the USA alone every year[1]. Compared to traditional sutured repair, hernia repair with meshes could obtain a more satisfactory postoperative effect[2], and tension-free repair with prosthetic mesh can reduce the risk of recurrence by 50 to 75%[3]. To achieve satisfactory outcomes, mesh repairs are adopted by more and more abdominal surgeons, especially for the repair of large inguinal hernias. Currently, tension-free repair with mesh is regarded as the gold standard for inguinal hernia repair, and mesh repair has replaced direct sutures. This increased mesh use has resulted in accelerated mesh production, with more than 200 mesh types available for hernia repair in the United States at present[4]. Currently, there are approximately 1 million prostheses used per year in the world[1].

Prosthetic meshes can be divided into synthetic and biologic types based on their sources of material. For several decades, polypropylene has been used worldwide in tension-free herniorrhaphy as a type of synthetic mesh. Polypropylene induces a strong foreign inflammatory reaction, then forms scar tissue to provide permanent repair to the abdominal wall defect with enough mechanical strength. In addition, polypropylene mesh is easy to handle and integrates quickly with the host; furthermore, with the use of polypropylene mesh, the post-operative recurrence rate can be reduced to under 1%[5]. However, synthetic meshes can lead to various complications, such as infection, intestinal fistula, intestinal obstruction, discomfort and chronic pain[6, 7]. Moreover, contamination is the relative contradiction to apply synthetic mesh, which would result in infection and repair failure[8].

Biological mesh is derived from humans and animals. Compared to synthetic materials, biological meshes can be absorbed by the host tissue and do not stimulate a continuous inflammatory reaction[9]. Small intestinal submucosa (SIS) is harvested from pigs and is subsequently transformed into biological mesh after a series process. SIS is the extracellular matrix compound collagen and some cell growth factors with other cellular components eliminated. SIS has several advantages, such as complete absorbability and good tissue compatibility; furthermore, SIS functions as a biologic scaffold for host tissues and cell reconstruction and growth[10]. Current studies have shown that SIS can be applied in inguinal hernia repair with good post-operative results[11, 12].

Numerous inguinal hernia repair techniques are currently available, and there is no exact standard. With the improvement of surgical techniques, laparoscopic surgery is widely used; however, the open surgical technique was shown to be superior to the laparoscopic technique for mesh repair of primary hernias in a large randomized clinical trial[13].

At present, there are only a few clinical trials to compare synthetic and biological meshes, and no consensus has been reached regarding which type is preferable for the repair of primary hernias. Polypropylene is the representative material in synthetic meshes, while SIS is the representative material in biological meshes. To compare polypropylene with SIS in open inguinal hernia repair, this study uses meta-analysis to systematically analyze randomized clinical trials to compare these meshes to determine whether the two materials generate any difference in recurrence, hematoma occurrence, seromas, or post-surgical pain and discomfort.

## Methods

### Search strategy

By performing a systematic search of MEDLINE, Embase and the Cochrane Library, we identified all articles published up to October 2014 using the search terms “inguinal or groin hernia”, “open repair”, “small intestinal submucosa”, “polypropylene”, “biologic mesh”, and “synthetic mesh”. Two authors carried out the search independently and compiled a reference lists of these primary studies to further identify trials of our interest. We scanned the abstracts of domestic and international conferences on hernia, finding no related RCTs

### Inclusion and exclusion criteria

We identified all clinical trials comparing porcine SIS with polypropylene in open inguinal hernia repair through an electronic database search. Included studies were required to meet the following criteria: 1. participants were adults with inguinal hernias, 2. The surgical technique was an open repair procedure, 3. SIS and polypropylene were chosen materials to compare, and 4. trials were randomized control trials (RCTs). Studies were excluded if they were case reports, reviews or duplicate publications, were performed on children or animals, or selected the laparoscopic inguinal hernia repair method. Non-randomized control trials or studies with incomplete information were also excluded.

### Data extraction

Two authors independently reviewed the included studies to extract the required data. The collected information contained 1. study characteristics (authors, date of publication, location of study, length of follow-up), 2. baseline characteristics (number of patients, mean age, gender, body mass index, type of intervention, type of mesh, type of technique, hernia details, operation time), and 3. outcome measures (infection, recurrence, hematomas, seromas, post-operation pain, discomfort). Based on the follow-up period and different observation points, post-operative pain was divided into two categories: acute pain within 30 days of surgery and chronic pain lasting for more than 1 year after surgery. Any disagreements were resolved by consensus.

### Quality assessment of included studies

We used the Cochrane Handbook for Systematic Reviews of Interventions version 5.2 from the Cochrane Collaboration guidelines[14] to assess the included RCTs’ risk of bias. The assessed bias included 1. random sequence generation (selection bias), 2. allocation concealment (selection bias), 3. blinding of participants and personnel (performance bias), 4. blinding of outcome assessment (detection bias), 5. incomplete outcome data (attrition bias), and 6. selective reporting (reporting bias) and other bias.

### Statistical analysis

The software Review Manager (Revman Version 5.2) provided by the Cochrane Collaboration was used for the statistical analysis to achieve a combined outcome. We used a fixed effect model to calculate pooled estimates of outcomes; however, a random effects model was also used based on heterogeneity. We used chi-square statistics to assess heterogeneity between trials and I^2^ statistic to assess the extent of inconsistency. Odds ratios (OR) with 95% confidence intervals (CIs) were calculated for the dichotomous data. Forest plots were used for the graphical display of results from the meta-analysis. We performed sensitivity analysis by removing individual studies from the data set and analyzing the overall effects size. Publication bias was assessed visually by evaluating the symmetry of funnel plots.

## Results

**Fig 1:**
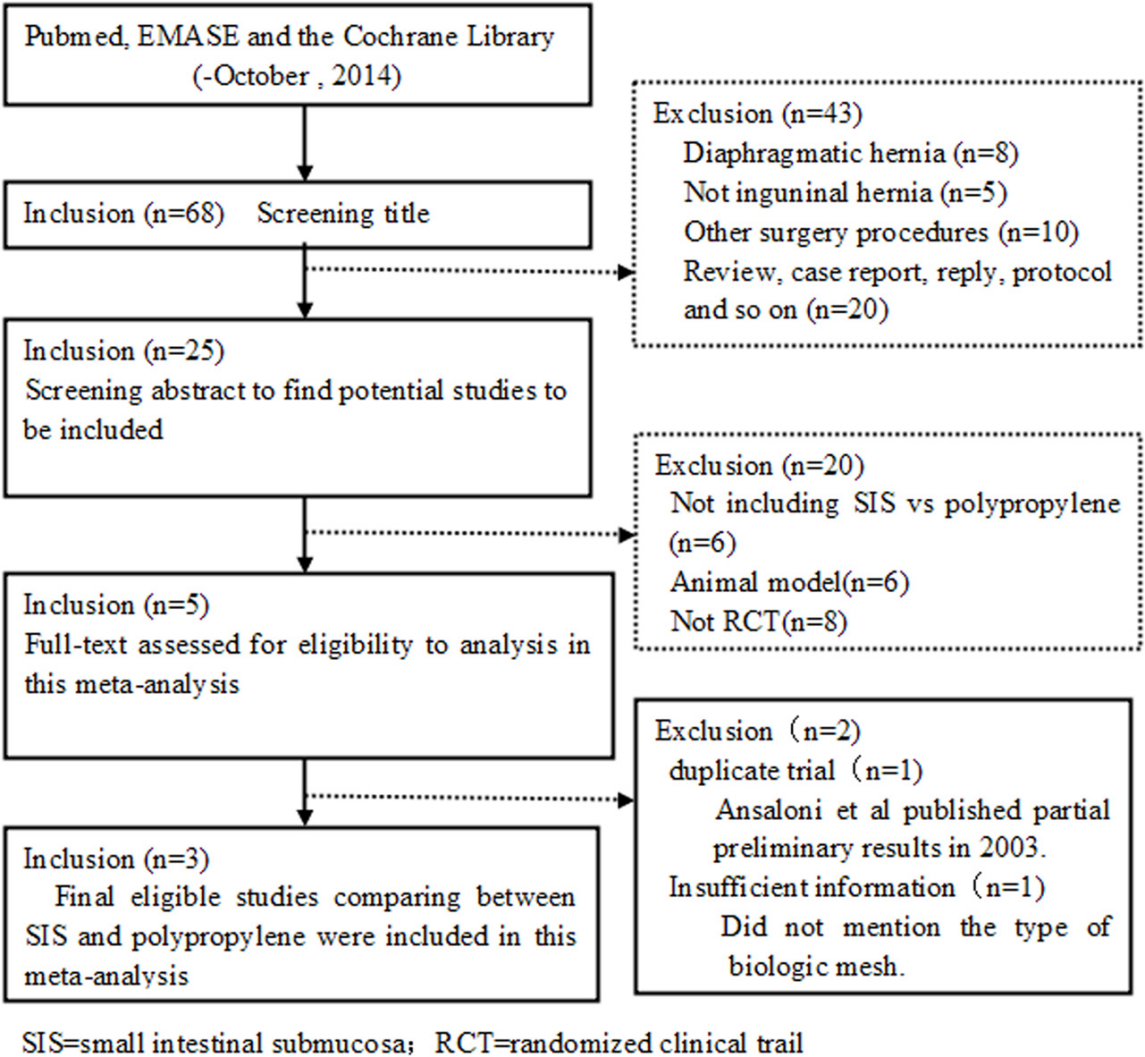
PRISMA flow diagram of the selection of studies.

The preferred reporting items for systematic reviews and meta-analyses flowchart of trial selection are shown in Fig 1. In total, three RCTs[15–17] encompassing 200 patients and comparing porcine SIS versus polypropylene in open inguinal hernia repair were selected for the meta-analysis. There were 100 patients in the SIS group and 100 patients in the polypropylene group. Although, there were only three studies in the meta-analysis, their baseline characteristics were exactly similar, as given in S1 and S2 Tables. The variables used for the meta-analysis are shown in S3 Table.

The quality assessment of the included studies shows the risk of bias (S4 Table). All included studies were randomized. Studies by Ansaloni et al.[16] and Bochicchio et al.[17] were both randomized double-blind trails; however, Puccio et al.[15] did not mention whether their trial was blind.

### Surgery time

All trials reported surgery times for the two groups, except for two trials[15, 17] that only calculated the mean time without providing the standard deviation (SD). We could not combine this variable in the meta-analysis; however, each of the three trials compared surgery times between the two groups and found that there was no significant difference.

### Recurrence

**Fig 2:**
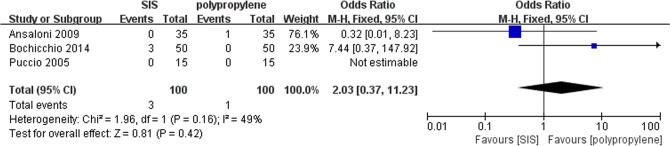
Forest plot of pooled odds ratio with 95% CI for comparing porcine small intestinal submucosa with polypropylene in open hernioplasty based on the assessment of recurrence.

All trials contributed to the combined calculation of recurrence. There was no significant heterogeneity (P = 0.16, I^2^ = 49%) among the three trials. In the fixed-effects model (OR = 2.03; 95% CI, 0.37 to 11.23; P = 0.4; Fig 2), there was no significant difference between the two groups in terms of recurrence after surgery.

### Hematomas

**Fig 3:**
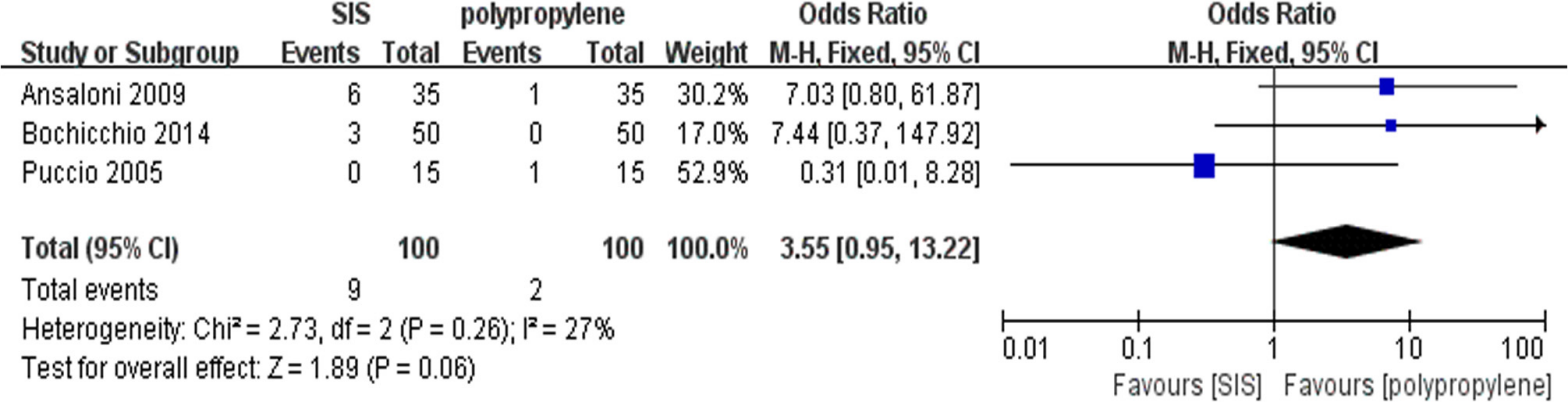
Forest plot of pooled odds ratio with 95% CI for comparing porcine small intestinal submucosa with polypropylene in open hernioplasty based on the assessment of hematomas.

All trials contributed to the combined calculation of hematomas. There was no significant heterogeneity (P = 0.26, I^2^ = 27%) among the trials. In the fixed-effects model (OR = 3.55; 95% CI, 0.95 to 13.22; P = 0.06; Fig 3), there was no significant difference between the two groups in terms of hematomas after surgery.

### Seroma

**Fig 4:**
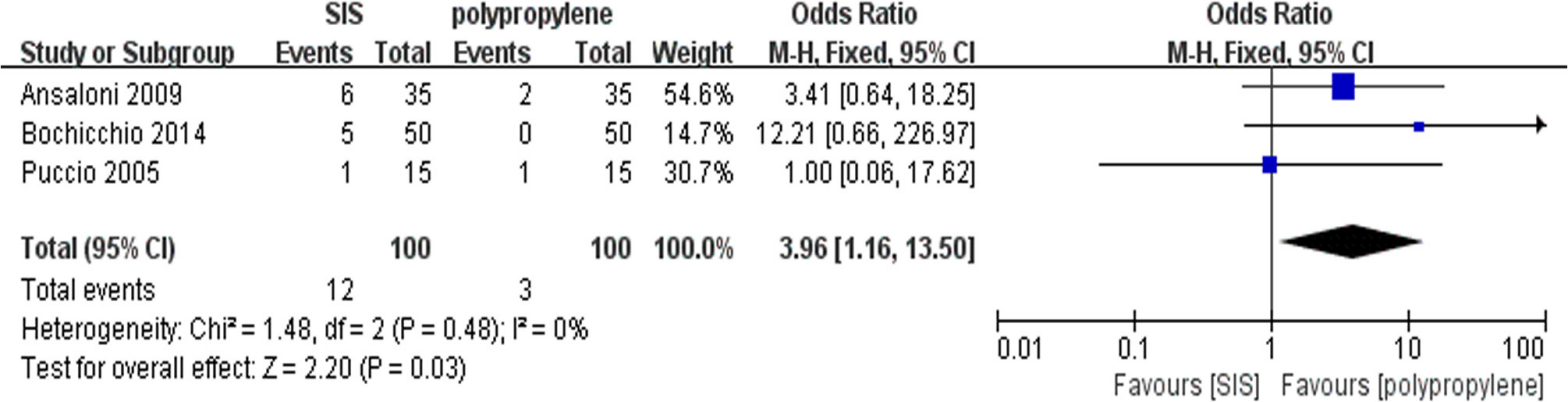
Forest plot of pooled odds ratio with 95% CI for comparing porcine small intestinal submucosa with polypropylene in open hernioplasty based on theassessment of seroma.

**Fig 5:**
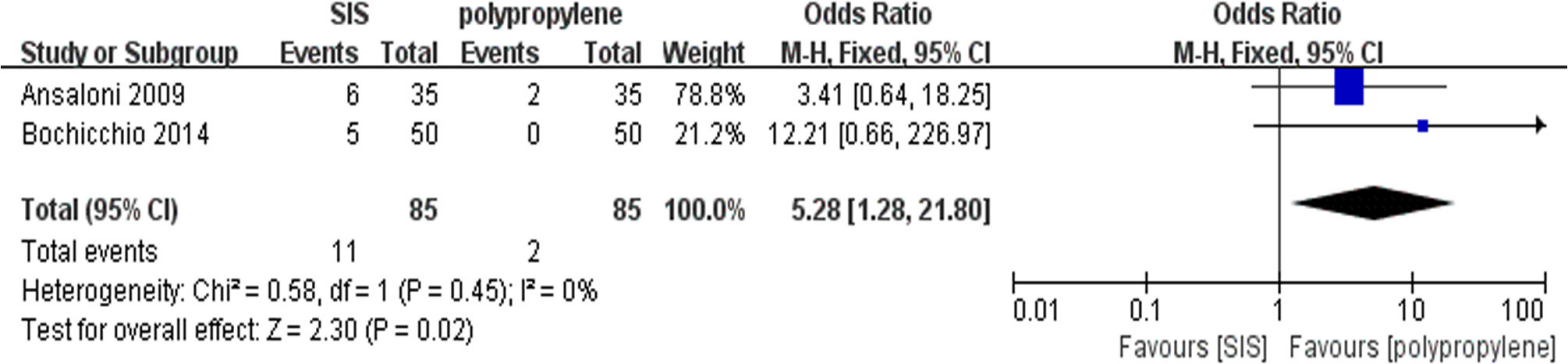
Forest plot of secondary analysis (2 RCTs) of the incidence of seroma after surgery.

**Fig 6:**
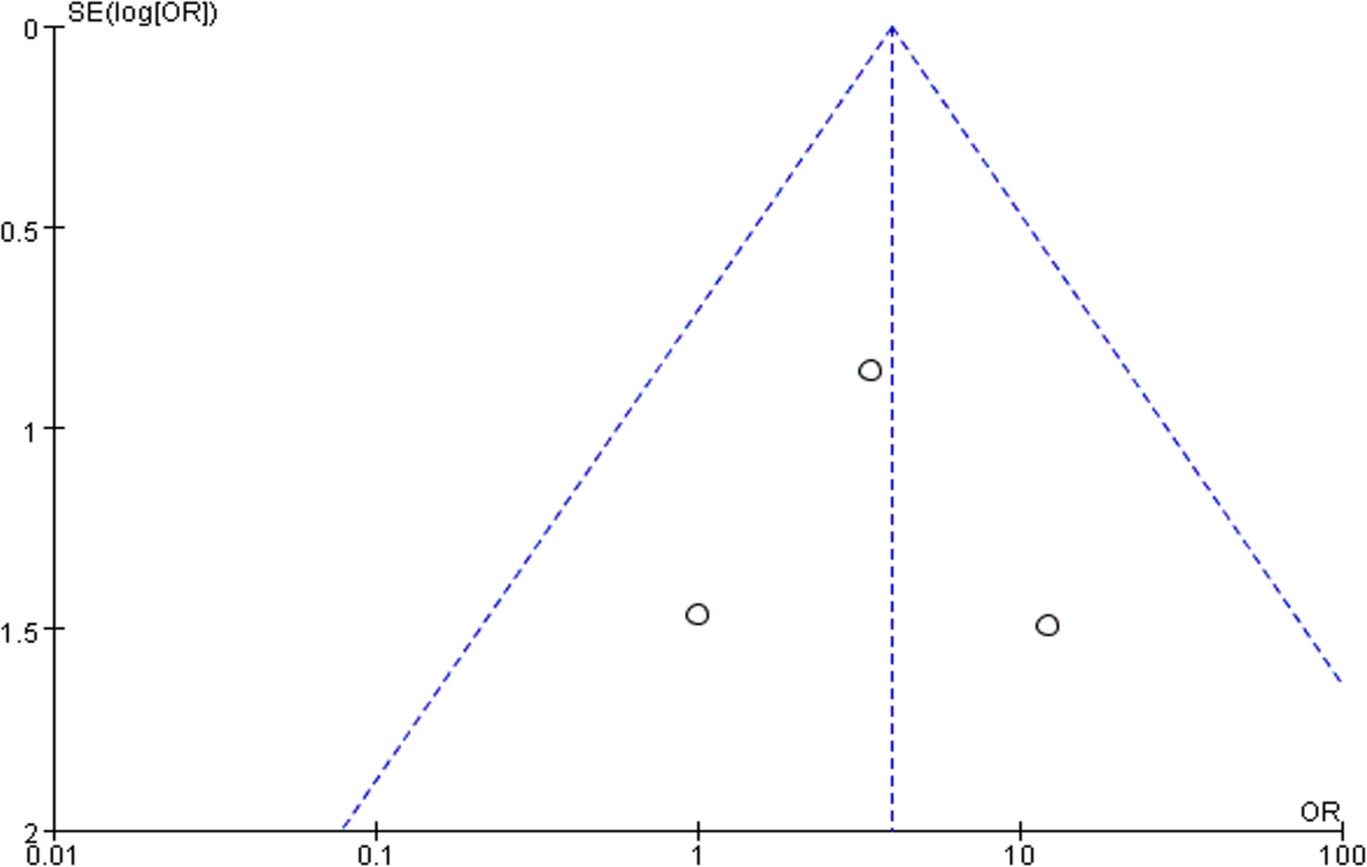
Funnel plot of the publication bias for incidence of seroma after surgery.

All trials contributed to the combined calculation of seroma. There was no heterogeneity (P = 0.48, I^2^ = 0%) among the trials. In the fixed-effects model (OR = 3.96; 95% CI, 1.16 to 13.50; P = 0.03; Fig 4), the incidence of seroma in the SIS group was higher than in the polypropylene group after surgery. To test the sensitivity of the result, we reanalyzed the incidence of seroma in 2 better quality RCTs by removing the trial by Puccio et al, and the same result was obtained (OR = 5.28; 95% CI, 1.28 to 21.80; P = 0.02; Fig 5). Visual assessment of the funnel plot does not suggest any publication bias(Fig 6).

### Post-operative pain in 30 days after surgery

**Fig 7:**
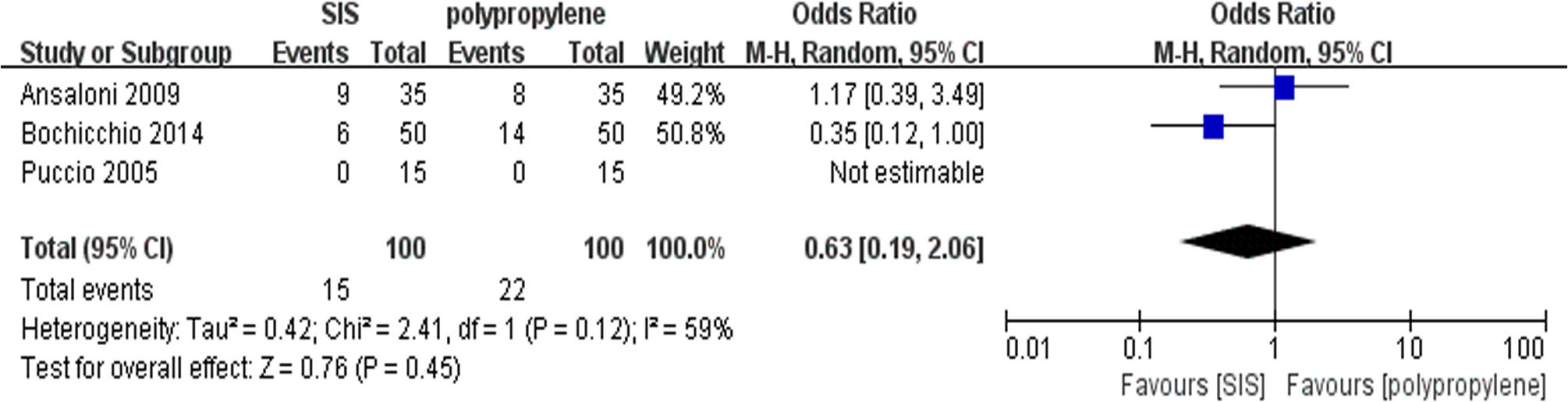
Forest plot of pooled odds ratio with 95% CI for comparing porcine small intestinal submucosa with polypropylene in open hernioplasty based on the assessment of post-operative pain in 30 days after surgery.

All trials contributed to the combined calculation of postoperative pain in 30 days after surgery. There was significant heterogeneity (P = 0.12, I^2^ = 59%) among the trials; therefore, we used the random-effects model (OR = 0.63; 95% CI, 0.19 to 2.06; P = 0.45; Fig 7). There were no significant difference in the incidence of post-operative pain in 30 days after surgery between the two groups.

### Post-operative pain within 1 year after surgery

**Fig 8:**
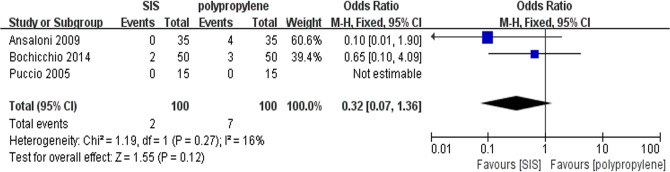
Forest plot of pooled odds ratio with 95% CI for comparing porcine small intestinal submucosa with polypropylene in open hernioplasty based on the assessment of post-operative pain within 1 year after surgery.

All trials contributed to the combined calculation of postoperative pain within 1 year of surgery. There was no significant heterogeneity (P = 0.27, I^2^ = 16%) among the trials. In the fixed-effects model (OR = 0.32; 95% CI, 0.07 to 1.36; P = 0.12; Fig 8), there were no significant differences between the two groups regarding the incidence of post-operative pain after 1 year.

### Discomfort

**Fig 9:**
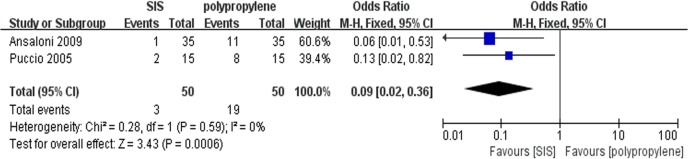
Forest plot of pooled odds ratio with 95% CI for comparing porcine small intestinal submucosa with polypropylene in open hernioplasty based on the assessment of discomfort.

Only two trials[15, 16] contributed to the combined calculation of discomfort after surgery. There was no heterogeneity (P = 0.59, I^2^ = 0%) among the trials. In the fixed-effects model (OR = 0.09; 95% CI, 0.02 to 0.36; P = 0.0006; Fig 9), the incidence of discomfort in the SIS group was lower than that in the polypropylene group after surgery.

## Discussion

At present, there are only few clinical trials comparing the results of small intestinal submucosa and polypropylene in open inguinal hernia repair, and large randomized trials do not exist. Therefore, we performed a meta-analysis to assess the efficacy of meshes made from the two materials. In total, 3 RCTs encompassing 200 patients were included in the meta-analysis. There was no difference in the baseline characteristics of the two groups in the study in terms of age, BMI and gender, and all trials used the Lichtenstein method to repair inguinal hernias. Our meta-analysis suggests that compared to polypropylene, using SIS in open inguinal hernia repair is associated with a lower incidence of discomfort, a higher incidence of seroma, and no significant differences in recurrence, hematoma occurrence and postoperative pain within 30 days or after 1 year of surgery.

Currently, synthetic meshes prepared from polypropylene and polytetrafluorethylene are widely used in clinical practice. However, there has been an increasing number of case reports on complications after surgery[6]. With the development of tissue engineering, biological materials have become available in clinical practice[18]. Small intestinal submucosa is one of the common biological materials in clinical practice. A preparation of small intestine was introduced for the replacement of vascular structures more than 40 years ago, and in the last decade, the FDA approved the clinical use of biological Inguinal Hernia Matrix (IHM; Cook Medical) derived from porcine small intestinal, which has been applied widely in clinical fields such as cardiology, dermatology, urology and general surgery[17, 19]. However, due to a lack of usage recommendations and guidelines, surgeons are only able to select meshes based on expert opinions and their own experience[20, 21].

In 1962, Usher first introduced the technique of tension-free hernia repair using synthetic meshes[22]. Since then, the quality of meshes and hernia repair techniques continue to improve. As a result, the recurrence rate has decreased significantly[23, 24]. Our study shows that there is no significant difference between the two groups in terms of recurrence after hernia operation.

However, another complication, namely, post-operative pain, especially chronic groin pain, is frequent. This chronic pain reduces the quality of life of patients and is becoming a common reason for litigation[25]. At present, a definition or consistent parameters to assess this pain are not available; therefore, chronic groin pain is currently described by the International Association as “groin pain reported by the patient at or beyond 3-months following inguinal hernia repair” [26]. In this study, the 3 included RCTs had different follow-up periods and data collection points; therefore, we assessed post-operative pain within 30 days and lasting for more than 1 year. The pain within 30 days did not include immediate pain after surgery. However, no significant difference was found between the incidence of post-operative pain within 30 days and that lasting for more than 1 year. Puccio et al.[15] used a visual analogue scale (VAS, 0–10) to record pain at 2 and 4 hours after surgery and confirmed less pain in the SIS group. Ansaloni et al.[16] measured pain at rest, on coughing, and during movement using a simple verbal scale (SVS) and a 100-mm visual analogue scale (VAS), showing that although there was no significant decrease in post-surgical pain incidence among patients in the SIS group, a significantly lower degree of pain was detected at rest and on coughing at 1, 3, and 6 months and during movement at 1, 3, and 6 months and 1, 2, and 3 years after surgery. polypropylene meshes are commonly made from plastic that triggers an inflammatory response and culminates in the formation of scar tissue[27, 28], which is likely to contribute to the chronic groin pain following inguinal herniorrhaphy. Unlike synthetic meshes, biological prosthesis are harvested from cadavers and animals and are processed physically, chemically and enzymatically to strip off the cellular material and antigens. The resultant prostheses consist of extracellular matrix, including collagen fibers, with various amounts of elastin and other proteins. Their ability to be remodeled allows the new materials to reduce post-inguinal herniorrhaphy chronic groin pain and are widely adopted by surgeons[3]. However, the elements causing chronic pain are still unclear, and there is no effective treatment available so far[29].

Several studies have demonstrated that biological meshes better tolerate contamination[30, 31]. In addition to the better tolerance to contamination, biological meshes are regarded to have a lower risk for adhesions, seroma, and encapsulation and less need for explantation when compared to synthetic meshes[4]. On the contrary, in our study, the incidence of seroma in the SIS group was higher than that in the polypropylene group after surgery; the SIS group also displayed a higher (albeit not significant) incidence of hematomas.

Badylak and Gilbert have shown xenogenic SIS to be non-immunogenic in vivo in clinical applications[32]. However, Ansaloni et al.[33] demonstrated that porcine SIS induced an immune response in humans. This immune response is associated with cell-associated galactose-alpha-1, 3-galactosyl-beta-1, and 4-N-acetyl glucosamine (a-gal)epitope expressed in primates and resulted in hyperacute rejection[34]. A major barrier to xenotransplantation in humans is the presence of natural antibodies to the terminal a-gal epitope, which is widely present in SIS[35, 36]. In animal models, implanting SIS to simulate a human alpha-Gal antigen reaction, there was no severe immune response; however, a slightly decelerated remodeling of the graft in mouse and rats was observed[37, 38]. In a primate model, a serum antibody response against alpha-Gal was detected with no other adverse effects[39]. Ansaloni et al.[33] used Surgisis for hernia repair in patients and obtained a similar result. Between 2–6 weeks, the alpha-Gal epitope showed a maximum response and then gradually decreased. In the study by Ansaloni et al.[16], all hematomas and seromas resolved without treatment within 3 months after surgery.

SIS is a biologic material with immunogenicity, which may induce more early immune responses than polypropylene in open inguinal hernia repair. However, in rat models, the results were opposite to our findings [40,41]. Therefore, our study provides a reference for abdominal surgeons, when they select meshes in open inguinal hernia. How to eliminate immunogenicity completely in biologic materials may become an area for future research.

There are several limitations to our study. First, although the baseline characteristics of the three trials were exactly similar, only 3 RCTs were included with a total of 200 patients. The small sample size makes our study not very persuasive. Second, there were differences in the inclusion and exclusion criteria among the included studies in terms of age, types of hernias, surgeon’s experience, etc. These differences may have influenced our results. Third, the included studies had differences in follow-up periods, observation points, measurement scales for post-operative pain, and definitions of chronic groin pain and other symptoms, which had an effect on evaluating post-operative pain and chronic pain directly. The trials with short follow-up periods did not provide enough time to observe and record postoperative complications. Lastly, there were potential deficiencies in date extraction in the trial by Ansaloni et al.[16], which may underestimate the effect of this study.

## Conclusions

Based on our study, it is suggested that compared to polypropylene, using SIS in open inguinal hernia repair is associated with a lower incidence of discomfort, a higher incidence of seroma, and no significant difference in recurrence, hematoma occurrence, and post-operative pain within 30 days or after 1 year of surgery. We need a standard definition for post-operative and chronic groin pain in patients undergoing inguinal hernia surgery and an internationally accepted pain measurement tool for homogeneous assessment. To confirm our findings and obtain further outcomes, well-designed larger RCTs with longer follow-up periods are needed.

## Supporting Information

S1 Appendix PRISMA Checklist. PRISMA guidance of the study.
(DOC)

S1 Table Basic information of the included trials. (DOC)

S2 Table Detailed information of the included trials. (DOC)

S3 Table Variables of included trials. (DOC)

S4 Table Risk of bias assessment of included trials. (DOC)
