# Supplementary material for: Comparison of Porcine Small Intestinal Submucosa versus Polypropylene in Open Inguinal Hernia Repair: A Systematic Review and Meta-Analysis
Source: PLoS One. 2015 Aug 7;10(8):e0135073. doi: 10.1371/journal.pone.0135073 (PMC4529205; doi:10.1371/journal.pone.0135073)
Supplement: S1 Table — (DOC) [file pone.0135073.s002.doc]

Table 1 Basic information of the included trials

| **Trial** | **Year** | **Country** | **Patients** | **Age(year)** | **BMI(kg/m^2^)** | **Gender** | **Type of mesh** | **Type of technique** | **Type of publication** | **Follow-up time(month）** |
| --- | --- | --- | --- | --- | --- | --- | --- | --- | --- | --- |
| Puccio et al | 2005 | Italy |  |  |  | male |  | Lichtenstein | RCT | 12（1-16） |
| SIS |  |  | 15 | 54(26-74) | 26 |  | Surgisis |  |  |  |
| polypropylene |  |  | 15 | 54(26-74) | 26 |  | Prolene |  |  |  |
| Ansaloni et al | 2009 | Italy |  |  |  | male |  | Lichtenstein | RCT | 36 |
| SIS |  |  | 35 | 56.2 ± 18.0 | 25.7±2.7 |  | Surgisis |  |  |  |
| polypropylene |  |  | 35 | 61.3 ±17.7 | 26.7±2.8 |  | PP |  |  |  |
| Bochicchio et al | 2014 | America |  |  |  | male |  | Lichtenstein | RCT | 12 |
| SIS |  |  | 50 | 64（24-85） | 26（18-39） |  | Surgisis |  |  |  |
| polypropylene |  |  | 50 | 59（25-87） | 25（19-37） |  | PP |  |  |  |

SIS=small intestinal submucosa；BMI=body mass index
