# Supplementary material for: Comparison of Porcine Small Intestinal Submucosa versus Polypropylene in Open Inguinal Hernia Repair: A Systematic Review and Meta-Analysis
Source: PLoS One. 2015 Aug 7;10(8):e0135073. doi: 10.1371/journal.pone.0135073 (PMC4529205; doi:10.1371/journal.pone.0135073)
Supplement: S2 Table — (DOC) [file pone.0135073.s003.doc]

Table 2 Detailed information of the included trials

| **Trial** | **Hernia details** | **Anesthesia** | **Surgeon performed operations** | **Sutures for mesh fixation** | **measurement scales** | **Radiological**  **examination** | **Observe points** |
| --- | --- | --- | --- | --- | --- | --- | --- |
| Puccio et al | Unilateral primary ingunal hernia | Local anesthesia | Five unblinded surgeons | 2-0 polypropylene （Prolene) | Visual analog scale(VAS,0-10) | Ultrasound，3months after surgery for every patient | 10 days,1-6months and 1 year |
| Ansaloni et al | Nocomplicated primary inguinal hernia | General or spine anesthesia | Two unblinded surgeons | Prolene3/0(Ethicon)  polydioxanone 2/0 | Simple verbal scale (SVS, include none, mild,moderate,severe, and unbearable) and visual analog scale(VAS,0-100) | Unstated | 1 week; 1 , 3, and 6 months; and 1 and 3  years |
| Bochicchio et al | Unilateral ingunal hernia | General or spine anesthesia | Surgeons,  blinding  unclear | Type of sutures not reported | Visual analog scale(VAS,0-10) and Short-Form 36 Health  Survey, version 2 (SF-36V2) | Ultrasound or CT scan，confirming a recurrence | 2 weeks, 3 months, and 1 year |
