# Supplementary material for: Comparison of Porcine Small Intestinal Submucosa versus Polypropylene in Open Inguinal Hernia Repair: A Systematic Review and Meta-Analysis
Source: PLoS One. 2015 Aug 7;10(8):e0135073. doi: 10.1371/journal.pone.0135073 (PMC4529205; doi:10.1371/journal.pone.0135073)
Supplement: S3 Table — (DOC) [file pone.0135073.s004.doc]

Table 3 Variables of included trials

| **Trial** | **Patients** | **Operation time（min)** | **Infection** | **Recurrence** | **Hematomas** | **Seromas** | **Postoperation pain(30 days)** | **Postoperation**  **pain(1 year)** | **Discomfort** |
| --- | --- | --- | --- | --- | --- | --- | --- | --- | --- |
| Puccio et al |  |  |  |  |  |  |  |  |  |
| SIS | 15 | 45（35-80） | 0 | 0 | 0 | 1 | 0 | 0 | 2 |
| polypropylene | 15 |  | 0 | 0 | 1 | 1 | 0 | 0 | 8 |
| Ansaloni et al |  |  | NG |  |  |  |  |  |  |
| SIS | 35 | 68.6 ±13.7  66±20.4 |  | 0 | 2 | 6 | 6 | 0 | 1 |
| polypropylene | 35 |  |  | 1 | 2 | 2 | 14 | 4 | 11 |
| Bochicchio et al |  |  |  |  |  |  |  |  | NG |
| SIS | 50 | 134  115 | 0 | 3 | 6 | 5 | 9 | 2 |  |
| polypropylene | 50 |  | 0 | 0 | 1 | 0 | 8 | 3 |  |

SIS=small intestinal submucosa；NG=not given
