# Supplementary material for: Comparison of Porcine Small Intestinal Submucosa versus Polypropylene in Open Inguinal Hernia Repair: A Systematic Review and Meta-Analysis
Source: PLoS One. 2015 Aug 7;10(8):e0135073. doi: 10.1371/journal.pone.0135073 (PMC4529205; doi:10.1371/journal.pone.0135073)
Supplement: S4 Table — (DOC) [file pone.0135073.s005.doc]

Table 4 Risk of bias assessment of included trials

|  | **Random sequence**  **generation**  **(selection bias)** | **Allocation**  **concealment**  **(selection bias)** | **Blinding of participants**  **and personnel**  **(performance bias)** | **Blinding of**  **outcome assessment**  **(detection bias)** | **Incomplete**  **outcome data**  **(attrition bias)** | **Selective**  **reporting**  **(reporting bias)** | **Other**  **bias** |
| --- | --- | --- | --- | --- | --- | --- | --- |
| **Puccio et al.** | **L** | **L** | **U** | **U** | **L** | **U** | **U** |
| **Ansaloni et al.** | **L** | **L** | **L** | **L** | **L** | **U** | **U** |
| **Bochicchio et al.** | **L** | **L** | **L** | **U** | **L** | **U** | **U** |

**L=low risk；H=high risk；U=unclear**
